# Louisville Summer School of Medicine

**Published:** 1845-08

**Authors:** 


### LOUISVILLE SUMMER SCHOOL OF MEDICINE.

The Lectures in the Louisville Summer School of Medicine will be resumed on the first Monday, which is the first day of September, and will continue till the last of October. Clinical lectures at the Hospital will constitute a portion of the course, and the usual facilities will be afforded to students for the study of anatomy. Students who propose availing themselves of the advantages of the lectures would do well to be present at the beginning. Y.

